# Association Between Academic, Cognitive and Health-Related Variables with Academic Stress in Health Sciences University Students

**DOI:** 10.3390/bs15091219

**Published:** 2025-09-08

**Authors:** Aniel Jessica Leticia Brambila-Tapia, Edgar Ulises Velarde-Partida, Laura Arely Carrillo-Delgadillo, Fabiola Macías-Espinoza, Saúl Ramírez-De los Santos

**Affiliations:** 1 Departamento de Psicología Básica, Centro Universitario de Ciencias de la Salud (CUCS), Universidad de Guadalajara, Sierra Mojada #950, Colonia Independencia, Guadalajara 44340, Mexico; ulises.velarde@academicos.udg.mx; 2 Licenciatura en Psicología, Centro Universitario de Ciencias de la Salud (CUCS), Universidad de Guadalajara, Guadalajara 44340, Mexico; laura.carrillo4391@alumnos.udg.mx; 3 Departamento de Psicología Aplicada, Centro Universitario de Ciencias de la Salud (CUCS), Universidad de Guadalajara, Guadalajara 44340, Mexico; fabiola.macias@academicos.udg.mx; 4 Instituto de investigación en Ciencias Biomédicas (IICB), Centro Universitario de Ciencias de la Salud (CUCS), Universidad de Guadalajara, Guadalajara 44340, Mexico

**Keywords:** academic stress, sex differences, academic stressors, procrastination

## Abstract

Academic stress arises from students facing academic demands and is linked to various academic and psychological factors. However, research has yet to explore its potential correlations with somatization, overall health issues, studying strategies, academic procrastination, academic performance, and intelligence scores. The objective of this study was to determine the potential correlations between such variables with academic stress in a sample of health sciences university students. University students of different bachelor’s programs were invited to participate; they fulfilled an electronic questionnaire with personal and psychological variables, including academic stress, and performed an intelligence test, which measures verbal and non-verbal intelligence. Finally, their academic achievement was measured with the grade point average (GPA). A total of 437 students were included, of which 296 (67.7%) were women, with a mean age of 20.36 ± 2.61 years old. Academic stress was higher in women than in men and showed moderate positive correlations with anxiety, depression, and somatization and a low positive correlation with the sum of diseases. It also showed a low negative correlation with sleep quality. In addition, academic stress correlated negatively with self-motivation, emotion perception, and emotion management as well as with active coping, positive relations with others, and the studying strategies (self-regulation, effort regulation, critical thinking, and time and study environment). We also observed a low positive correlation between academic stress and academic procrastination, which was higher in women than in men. No correlations were found with GPA or intelligence scores. In conclusion, academic stress was positively correlated with somatization, depression, anxiety, the sum of diseases, and academic procrastination; it was negatively correlated with emotional intelligence (mainly self-motivation), active coping, and specific studying strategies.

## 1. Introduction

Academic stress is defined as a systematic process of adaptive nature based in three stages: (a) the exposure of students to academic demands (academic stressors), (b) the appearance of systemic imbalance produced by the stressors (imbalance indicators), and (c) the taking of action by the student in order to respond to this imbalance (coping strategies) (Barraza-Macías, 2006; Guzmán-Castillo et al., 2018). Based on this theoretical model, a self-administered instrument was developed, which measures each one of these three stages (Barraza-Macías, 2007). Academic stressors have been associated with many factors including positive correlations with depression and somatization and negative correlations with the positive psychological variables—psychological well-being, life satisfaction, mindfulness—and personal variables, i.e., age and physical activity (Brambila-Tapia et al., 2019). In addition, students’ stress has been negatively correlated with students’ exam-related self-efficacy and rational and active coping strategies (Crego et al., 2016; Lopes & Nihei, 2021), while academic procrastination is another academic variable that has been positively correlated with academic stress (Khalid et al., 2019), which suggests that coping and studying strategies, as well as procrastination, are variables that also modify stress in students. With respect to health-related variables, perceived stress in students has been associated with the presence of chronic diseases and with the presence of physical symptoms (Barraza-Macías, 2007; Brambila-Tapia et al., 2019). In addition, the frequency of students with moderate to high perceived stress has been reported to be above 50% in Brazilian and Mexican student populations (Lopes & Nihei, 2021; Avila-Carrasco et al., 2023). With respect to sex differences, it has been shown that women have reported higher levels of academic stress than men, even if measured with different academic stress scales (Pérez-Jorge et al., 2025; Al-Shahrani et al., 2023; Shekhar & Kumar, 2016). In addition, negative correlations between academic stress and academic achievement have been shown (Bibi et al., 2022; Gustems-Carnicer et al., 2019; Crego et al., 2016; Deng et al., 2022), although these results are controversial, with another report showing opposite relationships (Al-Shahrani et al., 2023). Nevertheless, few studies investigating the relationship of different variables with academic stress may be found (Al-Shahrani et al., 2023; Brambila-Tapia et al., 2019). Therefore, research into the association of different factors with academic stress, including academic variables, is needed in order to better understand this phenomenon.

In this sense, despite the information reported in relation to academic stress and the many associated variables, to date, there are no studies that have assessed the possible relationship between academic stress and a wide range of personal, academic and psychological variables including academic procrastination, verbal and non-verbal intelligence, academic achievement, studying strategies, and health-related variables in university students. Therefore, the objectives of this study are (1) to perform sex comparisons of academic stress and the studied variables; (2) to assess bivariate correlations between academic stress and health-related variables, including somatization and the sum of diseases, and to determine whether these variables are associated with academic stress in university students after controlling for confounding factors; and (3) to assess bivariate correlations between academic stress and the academic variables (academic procrastination, studying strategies and academic achievement), thus determining whether these variables are associated with academic stress after controlling for confounding factors including verbal and non-verbal intelligence. The hypotheses of this study are as follows: (1) women have higher levels of academic stress than men; (2) there is a positive correlation between somatization and the sum of diseases and academic stress; (3) there is a positive correlation between procrastination and academic stress, and negative correlations between studying strategies and academic achievement with academic stress; and (4) the bivariate and multivariate correlations found differ by sex.

## 2. Subjects and Methods

### 2.1. Ethical Considerations

This study received approval from the ethical committee at the Health Sciences University Center under registration number CI-06022. It adhered to the guidelines outlined in the Declaration of Helsinki. All personal and health-related information was treated with the utmost confidentiality and only used for the purpose of this research. All participants signed informed consent.

### 2.2. Subjects

The inclusion criteria for participants were as follows: (a) being a student at the Health Sciences University Center of the University of Guadalajara; (b) being over 18 years old; and (c) signing informed consent. Participants were excluded if they did not complete all the measurements of the study.

### 2.3. Study Design

This is an observational and cross-sectional design, in which the objective of the study was to determine the association between the many independent variables and the dependent variable, academic stress.

### 2.4. Procedures

Students from different health science bachelor’s programs of the University of Guadalajara were invited to participate during their courses. Those who agreed were situated in a computer room of the University facilities. There, after signing informed consent, they fulfilled an electronic questionnaire including a wide range of sociodemographic and psychological variables. Afterwards, they performed an intelligence test.

### 2.5. Variables

The following variables were measured in the study.

### 2.6. Sociodemographic and Health Variable

The sociodemographic variables included sex, age, education level, maternal and paternal education level, number of siblings, daily minutes of physical activity, whether they had a romantic partner, whether they had a job, whether they had children, and the monthly money available for their expenses (in 5 categories ranging from nothing to more than USD 180 monthly). Additionally, the frequency of alcohol consumption, smoking, and illegal drug consumption were measured from never to many times in the week.

The presence of 21 chronic diseases and any additional disease in the last 6 months were measured (Brambila-Tapia et al., 2024). Sleep satisfaction was evaluated using the first item of the OVIEDO sleep questionnaire, with 7 response options, from very unsatisfied to very satisfied (Bobes-García et al., 2000). Sleep quality was measured with the second item (including 5 questions) of the same scale, ranging from 1 (low quality) to 5 (high quality) (Bobes-García et al., 2000).

### 2.7. Psychological Variables

Academic stress was assessed using the academic stressor subscale of the SISCO scale, which varied from 1 (low) to 5 (high) (Barraza-Macías, 2007; Guzmán-Castillo et al., 2018). Depression was measured with the PHQ-9 instrument, which varied from 1 (low) to 4 (high) (Baader et al., 2012), while anxiety was evaluated using the GAD-7 tool, from 1 to 4 (low to high) (García-Campayo et al., 2010). Somatization was assessed with the PHQ-15, ranging from 1 to 3 (low to high) (Ros-Montalbán et al., 2010). Active coping was measured through four items from the active coping component of the Mini-COPE scale and varied from 1 (low) to 4 (high) (Brambila-Tapia et al., 2023). The emotional intelligence questionnaire TEIQUE was used to assess subscales of emotional perception, emotional management, and self-motivation, each consisting of four to five items, and varied from 1 to 7 (totally disagree to totally agree) (Chirumbolo et al., 2019) (Supplementary File S1). Finally, we evaluated the subscale of positive relations with others from the shortened version of the psychological well-being scale, which varied from 1 to 5 (low to high) (Díaz et al., 2006).

### 2.8. Academic Variables and Intelligence

From the university records, we obtained the academic variables. These were grade point average (GPA), which served as the measurement for academic achievement, and preparatory GPA. Additionally, verbal (vocabulary) and non-verbal (abstraction) intelligence were assessed using the Spanish-validated version of the Shipley-2 instrument (Shipley et al., 2014). With this test, we obtained the verbal, non-verbal and the global (combined) punctuation.

### 2.9. Studying Strategies

The studying strategies measured from the Motivation Strategies for Learning Questionnaire (MSLQ) (Duncan & McKeachie, 2005) included seven learning strategy scales (time and study environment, critical thinking, rehearsal, effort regulation, self-regulation, elaboration, and organization) and two motivation scales (task value and intrinsic goal orientation), which were translated into Spanish and measured in the sample (see Supplementary File S2). In addition, the academic procrastination was assessed using the short version of the academic procrastination scale (Yockey, 2016) which was also translated into Spanish (refer to Supplementary File S3), all these scales varied from 1 (absolutely disagree) to 5 (absolutely agree).

### 2.10. Statistical Analysis

To describe the data, we used means and standard deviations for parametric quantitative variables, and medians and ranges for non-parametric ones. To describe qualitative variables, we used frequencies and percentages. In order to compare qualitative variables between 2 groups, we used the Chi-squared and Fisher’s exact test, while *T*-tests and Mann–Whitney U tests were used to compare quantitative variables between sexes, depending on the distribution of the data (parametric and non-parametric, respectively).

We studied the correlations between academic stress and other variables using Pearson and Spearman tests based on data distribution. To identify the independent variables most correlated with academic stress, we applied multiple linear regression with a step-wise method, detecting significant variables and reducing confusion bias. All scales proved reliable, with Cronbach alpha scores above 0.6.

## 3. Results

Out of all the invited participants, a total of 440 agreed to participate (around 60% of invited students). From these, 3 participants were excluded of the study for incomplete data, then the final sample consisted of 437 students, of whom 296 (67.7%) were women. The mean age (±SD) was 20.36 (±2.61) years, with a range of 18–54 years. The participants were enrolled in one of six different bachelor’s programs including medicine, psychology, and nursing.

Table 1 shows the descriptive results of the studied variables with the comparisons between sexes, while Table 2 shows the comparison between each academic stressor between sexes. Among the academic stressors of the SISCO scale, we observed that the overload of tasks and academic works, limited time to do the work, and the evaluations of professors had the highest scores and were significantly higher in women than in men; other stressors with higher values in women than in men were participation in class and the personality and character of professors. Most academic stressors showed a significantly higher frequency in women than in men (Table 2).

### 3.1. Bivariate Correlations Between the Studied Variables and Academic Stress

In Table 3, we show the significant correlations in the bivariate analysis between the studied variables and academic stress in the global sample and segmented by sex. In the global sample, we observed that male sex negatively correlated with academic stress (with a low correlation coefficient); the same was observed with daily free hours and daily physical activity minutes (with a very low correlation coefficient) and sleep quality (with a low correlation coefficient). In addition, academic stress positively correlated with somatization, anxiety and depression (with moderate correlation coefficients), and the sum of diseases (with a low correlation coefficient). With respect to the academic variables, academic stress correlated negatively with four studying strategies (time and study environment, effort regulation, self-regulation, and critical thinking) and positively with rehearsal, preparatory GPA, and academic procrastination. All these correlations, though significant, showed very low correlation coefficients. In addition, we identified negative correlations between academic stress and the positive psychological variables (i.e., emotion perception, emotion management and self-motivation (with low correlation coefficients)), and with active coping, and positive relations with others (with very low correlation coefficients).

### 3.2. Multivariate Analyses of Academic Stress

In the multivariate analysis of academic stress, it was found that in the global and women’s samples, self-motivation was the variable most correlated (negatively) with it; in addition, the sum of diseases was a variable positively correlated with academic stress in the three samples. The subscales of emotional intelligence emotion management and emotion perception were also negatively correlated with academic stress. Emotion management was negatively correlated with it in the global and women’s samples, and emotion perception was negatively correlated with academic stress in the global and men’s samples. In addition, the studying strategy organization was positively correlated with academic stress in the women’s sample, while elaboration was negatively correlated with it in the men’s sample, and academic procrastination was positively correlated with academic stress in the global and women’s samples (Table 4, Table 5 and Table 6). Additional correlations were found for personal variables in the three samples, where daily free hours, male sex and sleep quality were negatively correlated with academic stress in the global sample (Table 4). It is important to point out that the R of the three models obtained was moderate (~0.50), and the contribution of each variable to the multivariate models showed a low change in R^2^.

## 4. Discussion

In the present study, we searched for tentative correlations between a wide range of personal, academic, cognitive and psychological variables with academic stress in health sciences university students. We observed that in the sex comparisons of psychological variables, the women’s sample had higher levels of academic stress, despite a similar distribution from sociodemographic variables. These results coincide with previous reports, where anxiety, depression, perceived stress, the sum of diseases, and somatization were higher in women than in men (Ladwig et al., 2001; Vargas-Prada et al., 2016; Brambila-Tapia et al., 2022; Liguori et al., 2018). Therefore, the higher levels of academic stress in women than in men are related to the high frequency of anxiety and depression in this sex, which has been explained by hormonal factors, among which testosterone has been observed to be a protective hormone against anxiety and depression. It has also been observed that during times when women experiment hormonal flux—including puberty, menopause, perimenopause and post-partum periods—they are more likely to experience mood disturbance, anxiety, and depression (McHenry et al., 2014). However, other cultural and familiar factors related to higher expectations and demands in women could also be contributing to this difference. In this sense, women could benefit more from preventive and intervention programs intended to diminish academic stress, as this sex is at a higher risk. Concerning the main academic stressors reported, the three stressors with the highest values were similar to those found by Avila-Carrasco et al. (2023) in a sample of Mexican medical students during pandemics. These coincidences suggest that overload of tasks and academic work and the evaluations of professors are some of the main academic stressors in Mexican university students. The sex differences in academic and intelligence variables have already been discussed in a previous report by the research team of Brambila-Tapia et al. (2024).

With respect to the correlations, we observed that somatization, anxiety, depression, and the sum of diseases were the psychological and health-related variables that positively correlated with academic stress, with a higher correlation for anxiety, depression, and somatization, which showed a moderate correlation coefficient with academic stress. These results coincide with a previous report showing significant positive correlations between perceived stress and somatization and the sum of diseases and between academic stress and somatization, depression, and the sum of diseases (Brambila-Tapia et al., 2019, 2022). These results suggest that perceived stress and academic stress are correlated variables, which present a positive feedback with depression and anxiety, variables that are in turn correlated with somatization and the sum of diseases. This last association can be explained by the influence of stress and negative emotional states with inflammation and oxidative stress which could lead to disease development (Liguori et al., 2018; Furman et al., 2019). We also found that sleep quality and sleep satisfaction were negatively correlated with academic stress with a low correlation coefficient for both variables, a finding that coincides with a previous report of the research team showing that sleep quality negatively correlates with stress, depression, and anxiety (Brambila-Tapia et al., 2022). All these results regarding sex differences in academic stress along with health-related variables and academic stress corroborate the first and the second hypotheses of the study.

On the other hand, academic stress showed negative and very low correlations with having a job, daily free hours, and physical activity minutes. In the case of physical activity, these results coincide with our previous report (Brambila-Tapia et al., 2019), where academic stress presented a very low negative correlation with physical activity; in this report, we found a similar correlation value for the combined sample, and it was higher for the men’s sample, suggesting that male students could benefit more from exercise in order to diminish academic stress. However, larger and longitudinal studies assessing sex differences are needed in order to determine these correlations. With regard the negative correlation with daily free hours being higher in the women’s sample, this is an interesting finding that suggests that students with less academic load and/or those with more time to relax or be distracted show less academic stress, a correlation that could be more pronounced in women and which could also be related to the negative correlation between having a job and academic stress (given that having a job is related to distraction from academic activities as well as to other positive psychological variables). We did not find a previous report searching for these possible associations with which to compare these results.

With respect to psychological variables correlated with academic stress, we found that the three subscales of emotional intelligence (self-motivation, emotion perception and emotion management) showed very low negative correlations with academic stress, which were higher for self-motivation in women and for emotion perception in men. This suggests that an increase in emotional intelligence could diminish academic stress. These results coincide with a previous report by our research team where emotional clarity and repair negatively correlated with perceived stress in the Mexican general population (Macías-Espinoza et al., 2022) and with other reports showing a relationship between emotional intelligence and active coping (Downey et al., 2010). However, in this report, we also found that self-motivation could be related to a diminishment of academic stress, mainly in women. The importance of self-motivation is supported by the fact that this was the variable most correlated (negatively) with academic stress in the multivariate analysis of the global and women’s samples and could be explained by the protective effect that motivation for academic work could have on academic stress. These observations are in line with the self-determination theory, which postulates that three innate factors (competence, autonomy, and relatedness) need to be satisfied in order to enhance self-motivation and mental health, and when they are thwarted, motivation and well-being are diminished (Ryan & Deci, 2000). Therefore, in this study, we observed many variables related to emotional intelligence including self-motivation, emotional perception, and emotional management, along with variables related to psychological well-being—such as positive relationships with others—being negatively correlated with academic stress.

Other psychological variables negatively correlated with academic stress and with a very low correlation coefficient are positive relationships with others (a subscale of psychological well-being) and active coping; these results coincide with previous reports showing that active and rational coping are associated with less perceived stress in students (Crego et al., 2016; Lopes & Nihei, 2021) and with our previous research showing that positive relations with others presented a low negative correlation with academic stress. Other subscales of psychological well-being such as autonomy, purpose in life, environmental mastery, and self-acceptance have also been negatively correlated with academic stress in university students (Brambila-Tapia et al., 2019). The correlations observed in this study indicate that an increase in active coping and social support could diminish academic stress, although observational longitudinal or experimental studies should be performed in order to confirm this hypothesis.

With respect to the academic variables, we did not observe a correlation between GPA or intelligence with academic stress, and only a very low positive correlation between academic stress and preparatory GPA was detected; these results suggest that academic stress does not influence academic achievement in any sex and that academic stress is not influenced by intelligence scores (verbal or non-verbal) either globally or in a specific sex. Previous reports showing a negative correlation between academic stress and academic achievement have shown wide variability in the instruments used to measure academic stress; for instance, some of them only measure perceived stress with the perceived stress scale (PSS) and not academic stress (Gustems-Carnicer et al., 2019; Crego et al., 2016), while others used stress scales that—in addition to academic stressors—include aspects other than academic ones, such as familiar or environmental stressors (Bibi et al., 2022; Sahu et al., 2024; Deng et al., 2022). In addition, academic achievement was used as a measure with self-reported scales instead of GPA in some of these reports (Sahu et al., 2024; Deng et al., 2022), and those which included GPA to measure academic achievement correlated it with perceived stress and not with academic stress (Gustems-Carnicer et al., 2019; Crego et al., 2016). Interestingly, these two repots only detected very low negative correlations between these two variables (r = −0.116 and r = −0.13, respectively). In addition, one study (Al-Shahrani et al., 2023) which performed comparisons of segmented stressors found that in the domain of teaching and learning-related stressors, students with higher GPA showed higher levels of high stress, which suggests that the relationship between academic stress and academic achievement depends on the instruments used to measure these variables. However, by considering the very low positive correlation between preparatory GPA and academic stress, it is possible that a correlation between GPA and academic stress could exist, which could be detected with observational longitudinal or experimental studies. These correlations coincide with the aforementioned report showing that higher GPA scores are related to higher levels of teaching- and learning-related stressors (Al-Shahrani et al., 2023). Therefore, further studies will determine the accuracy of these findings.

We also found that academic procrastination showed a very low positive correlation with academic stress in the global and women’s samples, results that coincide with a report showing positive associations between procrastination and student stress (Khalid et al., 2019). In addition, procrastination levels were higher (with a borderline *p* value = 0.089) in men than in women, also coinciding with this previous report (Khalid et al., 2019). We found that this correlation was higher and significant only in the women’s sample, which suggests that academic procrastination may affect more women than men, a difference that could be also responsible for the higher levels of this behavior in male students.

We found that many studying strategies correlated negatively (showing very low correlation coefficients) with academic stress, including effort regulation, self-regulation, critical thinking, and time and study environment, while rehearsal showed a very low positive correlation with academic stress. These results are explained by the fact that the employment of these studying strategies, which are positively correlated with academic achievement (Credé & Phillips, 2011), could diminish academic stress by increasing academic self-efficacy, which has been negatively correlated with students’ stress (Crego et al., 2016). With respect to specific strategies and sex differences, we found that effort regulation and time and study environment showed higher negative correlations with academic stress in women than in men, while rehearsal also showed higher positive correlations in women than in men; these results suggest that the effort and time dedicated to study is more relevant to women than men in reducing academic stress, which could also be related to the higher correlation between procrastination and academic stress in women than in men and with the higher levels of academic stress in women. In addition, these results also suggest that rehearsal could be a maladaptive studying strategy; this could be explained by the fact that rehearsal consists of repeating and memorizing information rather than in understanding it.

The other investigated studying strategies that did not show a significant correlation with academic stress in the bivariate analysis did show it in the multivariate analyses, with organization being positively correlated with academic stress in the multivariate analysis for women and elaboration being negatively correlated with academic stress in the multivariate analysis for men, which indicates that these strategies can be correlated with academic stress after controlling for confounding variables. However, further studies with comparisons of sex difference are needed in order to corroborate these results. All these reported correlations between academic variables and academic stress permitted us to partially accept the third hypothesis, considering that academic procrastination positively correlated with academic stress and some (but not all) studying strategies negatively correlated with academic stress. In addition, academic achievement did not correlate with academic stress. With respect to the multivariate analyses, we observed that all the models showed moderate R values, indicating that these models explain the variability of academic stress to a low to moderate degree, which suggests that unmeasured variables are also predictors of academic stress.

These observations suggest that the implementation of mandatory courses and workshops intended to increase emotional abilities (including emotional intelligence and resilience) are needed for both professors and students in order to diminish academic stress in the university setting. In addition, the inclusion of techniques for increasing studying strategies and active coping in these courses, as well as social support, could diminish academic stress and its health-related consequences in university students. These workshops and courses should be tailored to the needs and the sex of students and professors, which would increase the efficacy of these intervention programs. In addition, these programs should be part of university policies in order to warrant their implementation and evaluation.

The main limitation of the study is its cross-sectional design, which does not permit us to determine causal relationships between variables. In addition, the men’s sample was smaller than the women’s sample which diminished the probability of finding significant correlations in this sex. However, the main strengths of this study are the inclusion of many personal, psychological, and academic variables which are related to academic stress but have not been previously investigated. In addition, the performance of sex comparisons and sex-segmented analyses permitted us to determine differences between sexes in the studied variables and specific correlations in each sex. Only two previous reports have been conducted in this manner in Mexican university students (Brambila-Tapia et al., 2019; Avila-Carrasco et al., 2023), and none of them have included the variables measured in the present study. In addition, this study adds new information to the few international studies performed to date with respect to academic stress.

In conclusion, we found that academic stress was significantly higher in women than in men and showed moderate positive correlations with anxiety, depression, and somatization, alongside a low correlation with the number of diseases; in addition, academic stress showed very low negative correlations with sleep satisfaction and sleep quality. The psychological variables of emotional intelligence, positive relations with others, and active coping showed low to very low negative correlations with academic stress. In addition, academic stress positively correlated (with a very low correlation coefficient) with academic procrastination and negatively correlated (with very low correlation coefficients) with four out of nine studying strategies studied, showing (very low) positive correlations with only two of these studying strategies. Finally, no correlations were found between academic achievement and verbal and non-verbal intelligence with academic stress. Further larger and longitudinal studies segmented by sex are needed in order to corroborate these results.

## Figures and Tables

**Table 1:** Descriptive data of sociodemographic, academic, psychological, and intelligence variables.

Variable	Combined Sample (n = 437)	Women (n = 296)	Men (n = 141)	*p* Value	º Effect Size
Age, median (range)	20.36 ± 2.61	20.30 ± 2.69	20.49 ± 2.41	0.513	
With job, n (%)	160 (36.6)	104 (35.1)	56 (39.7)	0.397	
With children, n (%)	6 (1.4)	6 (2.0)	0 (0.0)	0.189	
Socio economic level, n (%)				0.078	
- Very low	2 (0.5)	2 (0.7)	0 (0.0)		
- Low	68 (15.6)	41 (13.9)	27 (19.1)		
- Medium	345 (78.9)	242 (81.7)	103 (73.1)		
- High	22 (5.0)	11 (3.7)	11 (7.8)		
Schooling, n (%)				1.000	
- Secondary	2 (0.5)	1 (0.3)	1 (0.7)		
- Preparatory	408 (93.3)	277 (93.6)	131 (92.9)		
- University (Bachelor’s degree)	27 (6.2)	18 (6.1)	9 (6.4)		
University grade point average (GPA), median (range)	95.2 (75.4–99.9)	95.35 (80.7–99.7)	94.64 (75.4–99.9)	0.108	
Preparatory grade point average (GPA), median (range)	93.0 (71.0–100.0)	93.7 (71–100)	92 (75–100)	0.006 *	0.161
Verbal intelligence (Standard score), median (range)	106.0 (72.0–125.0)	106 (72–120)	108.5 (79–125)	0.019 *	0.139
Non-verbal intelligence (Standard score), median (range)	101.0 (73.0–130.0)	100 (76–124)	104 (73–130)	0.008 *	0.156
Combined intelligence (Standard score), median (range)	110.0 (82.0–129.0)	109 (82–127)	112 (82–129)	0.001 *	0.188
Academic procrastination, median (range)	2.8 (1.0–5.0)	2.8 (1.0–5.0)	3.0 (0.00–5.00)	0.089	
Time and study environment, median (range)	3.5 (1.8–5.0)	3.50 (1.75–5.00)	3.44 (1.75–4.88)	0.893	
Critical thinking, median (range)	3.4 (1.0–5.0)	3.20 (1.00–5.00)	3.60 (1.40–5.00)	0.001 *	0.201
Task value, median (range)	4.5 (1.0–5.0)	4.50 (1.67–5.00)	4.33 (1.00–5.00)	0.006 *	0.162
Rehearsal, median (range)	3.5 (1.0–5.0)	3.50 (1.25–5.00)	3.25 (1.00–5.00)	0.002 *	0.185
Effort regulation, median (range)	3.8 (1.3–5.0)	3.75 (1.25–5.00)	3.75 (1.75–5.00)	0.281	
Self-regulation, median (range)	3.6 (1.6–5.0)	3.57 (1.57–5.00)	3.57 (2.29–4.86)	0.459	
Elaboration, mean ± SD	3.5 (1.0–5.0)	3.49 ± 0.87	3.55 ± 0.80	0.575	
Intrinsic goal orientation, median (range)	4.0 (1.0–5.0)	4.00 (1.75–5.00)	4.00 (1.00–5.00)	0.207	
Organization, median (range)	3.5 (1.0–5.0)	3.50 (1.00–5.00)	3.25 (1.00–5.00)	0.004 *	0.169
Daily physical activity minutes, median (range)	60 (0–450)	60 (0–450)	60 (0–240)	0.001 *	0.153
Daily free hours, median (range)	3 (0–12)	3 (0–12)	3 (0–9)	0.865	
Number of siblings	2 (0–7)	2 (0–7)	2 (0–7)	0.993	
Sum of diseases, median (range)	3 (0–3)	3 (0–13)	2 (0–11)	0.001 *	0.241
Sleep quality (OVIEDO scale), median (range)	3.4 (1–5)	3.4 (1–5)	3.6 (1–5)	0.041 *	0.12
Frequency of illegal drug consumption, median (range) of the average of consumption of the 8 drugs evaluated	0 (0–1)	0 (0–0.88)	0 (0–1)	0.084	
Somatization (PHQ-15), median (range)	1.6 (1.0–2.9)	1.78 (1.07–2.86)	1.50 (1–2.36)	<0.001 *	0.382
Active coping (Mini-COPE), median (range)	3.0 (1.2–4.0)	3.00 (1.20–4.00)	3.20 (1.60–4.00)	0.092	
Emotion perception, mean ± SD	4.52 ± 1.53	4.38 ± 1.59	4.83 ± 1.35	0.002 *	0.155
Self-motivation, mean ± SD	4.84 ± 1.17	4.81 ± 1.18	4.88 ± 1.15	0.543	
Emotion management, mean ± SD	4.43 ± 1.21	4.21 ± 1.18	4.88 ± 1.14	<0.001 *	0.333
Depression (PHQ-9), median (range)	2.1 (1.0–4.0)	2.22 (1.00–4.00)	2.00 (1.00–3.89)	0.024 *	0.133
Anxiety (GAD-7), median (range)	2.3 (1.0–4.0)	2.43 (1.00–4.00)	2.00 (1.00–4.00)	<0.001 *	0.287
Positive relations with others, median (range)	3.8 (1.0–5.0)	3.80 (1.00–5.00)	3.60 (1.20 –5.00)	0.425	

Comparisons between sexes for quantitative variables were performed with the *T*-test and Mann–Whitney U test, depending whether the distribution was parametric or non-parametric, respectively. Comparisons between qualitative variables were performed with the Chi-squared and Fisher’s exact tests. * Statistically significant values (*p* < 0.05); º effect size was calculated only for significant *p* values, and this was calculated with the rank biserial correlation when the Mann–Whitney U test was performed and with Cohen’s r test when the *T*-test was performed. The possible values of GPA and preparatory GPA are ≤100. The possible values for all intelligence scores are ≤145.

**Table 2:** Sex comparisons of the frequency of the presence of each academic stressor.

Variable	Combined Sample (n = 437)		Women (n = 296)		Men (n = 141)		*p* Value	Effect Size
Academic stress (SISCO), median (range)	3 (1.0–4.9)		3.25 (1.4–4.8)		2.75 (1.0–4.8)		<0.001 *	0.276
**Type of stressor, n (%)**	**Low**	**High**	**Low**	**High**	**Low**	**High**	***p* value**	
Competition among classmates	384 (87.9)	53 (12.1)	253 (85.5)	43 (14.5)	131 (92.9)	10 (7.1)	0.284	
Overload of tasks and academic works	197 (45.1)	240 (54.9)	115 (38.9)	181 (61.1)	82 (58.2)	59 (41.8)	<0.001	
Personality and character of professors	348 (79.6)	89 (20.4)	232 (78.4)	64 (21.6)	116 (82.3)	25 (17.7)	0.007	
Evaluations of professors (exams, homework, essays, conceptual maps, etc.)	198 (45.3)	239 (54.7)	119 (40.2)	177 (59.8)	79 (56.0)	62 (44.0)	0.001	
Type of work that professors request	303 (69.3)	134 (30.7)	198 (66.9)	98 (33.1)	105 (74.5)	36 (25.5)	0.168	
Not understanding the topics covered in class	294 (67.3)	143 (32.7)	192 (64.9)	104 (35.1)	102 (72.3)	39 (27.7)	0.077	
Participation in class (e.g., response to questions, expositions, etc.)	298 (68.2)	139 (31.8)	186 (62.8)	110 (37.2)	112 (79.4)	29 (20.6)	<0.001	
Limited time to do work	238 (54.5)	199 (45.5)	154 (52.0)	142 (48.0)	84 (59.6)	57 (40.4)	0.003	

Low frequency was obtained by the sum of the options (never, rarely, and sometimes), and high frequency was obtained by the sum of almost always and always. Effect size (assessed with the rank biserial correlation) only applied for the SISCO global scale. * *p* values were obtained with the Mann–Whiney U test for the SISCO global scale and with the Chi-squared and Fisher’s exact tests for the comparison of each stressor between sexes.

**Table 3:** Significant bivariate correlations between the studied variables and academic stress.

Variable	Women (n = 296)	Men (n = 141)	Global Sample (n = 437)
Sex (Women = 1, Men = 2)	-	-	−0.224 **
Daily free hours	−0.201 **	0.003	−0.133 **
Having a job	−0.028	−0.167 *	−0.074
Daily physical activity minutes	−0.087	−0.203 *	−0.141 **
Sleep satisfaction	−0.109	−0.167 *	−0.109
Sleep quality	−0.240 **	−0.242 **	−0.267 **
Somatization	0.436 **	0.411 **	0.471 **
Sum of diseases	0.203 **	0.216 **	0.251 **
Schooling	−0.126 *	−0.102	−0.056
Preparatory GPA	0.089	0.146	0.135 **
Academic procrastination	0.250 **	0.075	0.177 **
Rehearsal	0.100	0.049	0.111 *
Time and study environment	−0.215 **	−0.102	−0.173 **
Critical thinking	−0.105	−0.116	−0.134 **
Effort regulation	−0.220 **	−0.132	−0.179 **
Self-regulation	−0.178 **	−0.156	−0.171 **
Active coping	−0.142 *	−0.075	−0.131 **
Emotion management	−0.236 **	−0.235 **	−0.275 **
Self-motivation	−0.308 **	−0.229 **	−0.271 **
Emotion perception	−0.248 **	−0.302 **	−0.276 **
Positive relations with others	−0.149 *	−0.170 *	−0.158 **
Anxiety	0.462 **	0.226 **	0.506 **
Depression	0.480 **	0.499 **	0.494 **

* *p* < 0.05, ** *p* < 0.01. *p* values obtained with Spearman correlation test.

**Table 4:** Multivariate regression analysis for the global sample.

Variable	B	Beta Coefficient	*p* Value	Tolerance	Change in R^2^
Constant	3.987	-	0.000	-	-
Self-motivation	−0.084	−0.126	0.033	0.577	0.088
Sum of diseases	0.040	0.114	0.020	0.845	0.042
Emotion management	−0.062	−0.097	0.078	0.673	0.025
Rehearsal	0.110	0.122	0.011	0.878	0.020
Daily free hours	−0.061	−0.155	0.001	0.922	0.014
Having a job	−0.195	−0.119	0.011	0.921	0.012
Academic Procrastination	0.077	0.118	0.024	0.738	0.010
Male sex	−0.169	−0.100	0.037	0.874	0.009
Emotion perception	−0.054	−0.105	0.047	0.715	0.008
Sleep quality	−0.075	−0.099	0.054	0.760	0.007

R of the model: 0.484. The variables anxiety, depression, and somatization were not included in the analysis because we consider that these are a consequence rather than a cause of academic stress.

**Table 5:** Multivariate regression analysis for the women’s sample.

Variable	B	Beta Coefficient	*p* Value	Tolerance	Change in R^2^
Constant	4.870	-	0.000	-	-
Self-motivation	−0.146	−0.220	0.001	0.648	0.107
Daily free hours	−0.093	−0.243	0.000	0.895	0.041
Emotion management	−0.108	−0.165	0.005	0.840	0.025
Schooling	−0.359	−0.111	0.049	0.920	0.020
Academic Procrastination	0.102	0.160	0.012	0.730	0.016
Organization	0.139	0.174	0.003	0.854	0.016
Having a job	−0.168	−0.102	0.083	0.836	0.008
Sum of diseases	0.031	0.092	0.098	0.934	0.008

R of the model: 0.502. The variables anxiety, depression, and somatization were not included in the analysis by considering that these are a consequence rather than a cause of academic stress.

**Table 6:** Multivariate regression analysis for the men’s sample.

Variable	B	Beta Coefficient	*p* Value	Tolerance	Change in R^2^
Constant	4.016	-	0.000	-	-
Sum of diseases	0.102	0.281	0.001	0.965	0.103
Emotion perception	−0.130	−0.241	0.005	0.908	0.065
Number of siblings	−0.130	−0.188	0.020	0.988	0.030
Illegal drug consumption	−0.102	−0.181	0.027	0.964	0.028
Elaboration	−0.146	−0.157	0.060	0.929	0.023

R of the model: 0.499. The variables anxiety, depression, and somatization were not included in the analysis because we consider that these are a consequence rather than a cause of academic stress.

## Data Availability

The data presented in this study are available on request from the corresponding authors.

## Supplementary Materials

The following supporting information can be downloaded at https://www.mdpi.com/article/10.3390/bs15091219/s1.

## Disclaimer

All authors confirm that the following manuscript is a transparent and honest account of the reported research. This research is related to a previous study by the same authors, entitled “Correlation between studying strategies and psychological factors with academic achievement and intelligence in health sciences university students: a cross-sectional study” (Brambila-Tapia et al., 2024). This previous study focused on variables related to academic achievement and intelligence in the same population; however, the current submission focuses on factors related to academic stress. This study follows a very similar methodology; therefore, some parts of the methodology are quite similar in both studies (including ethical considerations, eligibility criteria, study design, procedures, the main variables included, and the descriptive results), given that they were performed in the same population. For more details, please see the previous publication (Brambila-Tapia et al., 2024). However, the objectives of both studies are quite different; therefore, the Introduction, Results, and Discussion sections clearly differ between both studies.
