# Supplementary material for: Association Between Academic, Cognitive and Health-Related Variables with Academic Stress in Health Sciences University Students
Source: Behav Sci (Basel). 2025 Sep 8;15(9):1219. doi: 10.3390/bs15091219 (PMC12466569; doi:10.3390/bs15091219)
Supplement: Supplementary file 1 [file behavsci-15-01219-s001.zip › behavsci-3727433-supplementary.pdf]

Supplementary File S1

Items included in the emotional intelligence subscales of the TEIQUE scale

<u>Instruction</u>: Please indicate the degree of agreement or disagreement with the following statements. <u>Answer options</u>: 1: absolutely disagree- 7: absolutely agree

Self-motivation

1.- On the whole, I'm a highly motivated person (En general soy una persona con alta motivación)

2.- Sometimes, it feels like I'm producing a lot of good work effortlessly (Me cuesta trabajo esforzarme ante una tarea)

3.- I normally find it difficult to keep myself motivated (Me cuesta motivarme por lo que hago)

4.- I tend to get a lot of pleasure just from doing something well (Tiendo a obtener una gran satisfacción simplemente con hacer algo bien)

5.- I lose interest in what I do quite easily (Suelo perder interés por lo que hago)

Cronbach alpha = 0.689

Emotion regulation

1.- I tend to get "carried away" easily (Tiendo a enfadarme o exaltarme con facilidad)

2.- I usually find it difficult to regulate my emotions (Me cuesta controlar mis emociones)

3.- I´m usually able to calm down quickly after I´ve got mad at someone (Suelo calmarme con rapidez después de haberme enfadado con alguien)

4.- I´m usually able to find ways to control my emotions when I want to (Puedo encontrar diferentes maneras de controlar mis emociones cuando lo deseo)

5.- I know how to snap out my negative moods (Sé cómo animarme cuando me siento mal)

6.- When someone offends me, I´m usually able to remain calm (Cuando alguien me ofende soy capaz de mantener la calma)

Cronbach alpha: 0.733

Emotion perception

1.- I often find it difficult to recognize what emotion I am feeling (Me cuesta reconocer mis propias emociones)

2.- I´m never really sure what I´m feeling (Nunca estoy realmente seguro/a de lo que estoy sintiendo)

3.- Many times, I can´t figure out what emotion I´m feeling (Muchas veces no consigo tener claro qué emoción estoy sintiendo)

4.- On the whole, I find it difficult to describe my feelings (En general me resulta difícil describir mis sentimientos)

5.- Most of the time, I know exactly why I feel the way I do (La mayoría de las veces sé exactamente por qué siento lo que estoy sintiendo en ese momento)

Cronbach alpha:0.850

Supplementary File S2. MSLQ items included, English/Spanish

Instructions: please indicate the degree of agreement or disagreement with the following statements. Answer options: 1 = Absolutely disagree to 5 =Absolutely agree

Motivation Strategies for Learning Questionnaire

Task value

1.- I think I will be able to use what I learn in this course in other courses/ “Creo que puedo usar en otros cursos lo que aprendo en esta clase” (item 4)

2.- It is important for me to learn the course material in this class/ “Es importante para mí aprender la información de mis clases” (item 10)

3.- I am very interested in the content area of this course/ “Estoy muy interesado en el contenido de mis clases” (item 17)

4.- I think the course material in this class is useful for me to learn/ “Creo que la información de mis clases es útil para que la aprenda” (item 23)

5.- I like the subject matter of this course/ “Me gusta el tema de mis clases” (item 26)

6.- Understanding the subject matter of this course is very important to me/ “Es muy importante para mí comprender la información de mis clases” (item 27)

Cronbach alpha = 0.863

Rehearsal

1.- When I study for this class, I practice saying the material to myself over and over/ “Cuando estudio para mis clases practico diciendo la información varias veces” (Item 39)

2.- When studying for this class, I read my class notes and the course readings over and over again/ “Cuando estudio para mis clases leo las notas y lecturas varias veces” (item 46)

3.- I memorize key words to remind me of important concepts in this class/ “Memorizo palabras clave para recordar conceptos importantes de mis clases” (item 59)

4.- I make lists of important terms for this course and memorize the lists/ “Hago listas de términos importantes para mis clases y los memorizo” (item 72)

Cronbach alpha: 0.756

Time and study environment

1.- I usually study in a place where I can concentrate on my course work/ “Normalmente estudio en un lugar en donde puedo concentrarme en mi trabajo de clase” (item 35)

2.- I make good use of my study time for this course/ “Hago un buen uso de mi tiempo de estudio para mis clases” (item 43)

3.- I find it hard to stick to a study schedule/ “Me resulta difícil cumplir con un horario de estudio” (Reversed) (item 52)

4.- I have a regular place set aside for studying/ “Tengo un lugar fijo reservado para estudiar” (item 65)

5.- I make sure I keep up with the weekly readings and assignments for this course/ “Me aseguro de mantenerme al día con las lecturas y tareas semanales de mis clases” (item 70)

6.- I attend class regularly/ “Asisto a clases regularmente” (item 73)

7.- I often find that I don´t spend very much time on this course because of other activities/ “A menudo encuentro que no le dedico mucho tiempo a mis clases debido a otras actividades” (Reversed) (item 77)

8.- I rarely find time to review my notes or readings before an exam/ “Rara vez encuentro tiempo para revisar mis notas o lecturas antes de un examen” (Reversed) (item 80)

Cronbach alpha = 0.731

Critical thinking

1.- I often find myself questioning things I hear or read in this course to decide if I find them convincing/ “Frecuentemente me encuentro cuestionando cosas que escucho o leo en clase para decidir si me parecen convincentes” (item 38)

2.- When a theory, interpretation, or conclusion is presented in class or in the readings, I try to decide if there is good supporting evidence/ “Cuando se presenta una teoría, interpretación o conclusión en clase, trato de decidir si hay buena evidencia de apoyo” (item 47)

3.- I treat the course material as a starting point and try to develop my own ideas about it/ “Tomo el material de clase como punto de partida y trato de desarrollar mis propias ideas sobre el tema” (item 51)

4.- I try to play around with ideas of my own related to what I am learning in this course/ “Trato de jugar con mis propias ideas relacionadas con lo que estoy aprendiendo en clase” (item 66)

5.- Whenever I read or hear an assertion or conclusion in this class, I think about possible alternatives/ “Cada vez que escucho una afirmación o conclusión en clase pienso en posibles alternativas” (item 71)

Cronbach alpha = 0.804

Effort regulation

1.- I often feel so lazy or bored when I study for this class that I quit before I finish what I planned to do/ “A menudo me siento con tanta flojera o aburrimiento cuando estudio para mis clases que dejo de hacerlo antes de lo planeado” (item 37)

2.- I work hard to do well in this class even if I don't like what we are doing / “Trabajo duro para que me vaya bien en las clases, incluso si no me gusta lo que estamos haciendo” (item 48)

3.- When course work is difficult, I give up or only study the easy parts/ “Cuando el trabajo de las clases es difícil, me doy por vencido y solo estudio las partes fáciles” (item 60)

4.- Even when course materials are dull and uninteresting, I manage to keep working until I finish/ “Incluso cuando los temas del curso son aburridos o poco interesantes, sigo trabajando hasta que termino” (item 74)

Cronbach alpha = 0.689

Meta cognitive Self-regulation

1.- When I become confused about something I'm reading for this class, I go back and try to figure it out/ “Cuando me confundo con algo que estoy leyendo para las clases, regreso al punto y trato de resolverlo” (item 41)

2.- If course materials are difficult to understand, I change the way I read the material/ “Si los temas del curso son difíciles de entender cambio la forma en que leo el material” (item 44)

3.- Before I study new course material thoroughly, I often skim it to see how it is organized/ “Antes de estudiar a fondo nueva información, la hojeo para ver cómo está organizada” (item 54)

4.- I ask myself questions to make sure I understand the material I have been studying in this class/ “Me hago preguntas para asegurarme de que entiendo el material que he estado estudiando en clase” (item 55)

5.- I often find that I have been reading for class but don't know what it was all about/ “A menudo me encuentro que he estado leyendo para la clase, pero no sé de qué se trata” (Reversed) (item 57)

6.- When studying for this course I try to determine which concepts I don't understand well/ “Cuando estudio trato de determinar qué conceptos no entiendo bien” (item 76)

7.- During class time I often miss important points because I'm thinking of other things/ “Durante las clases, seguido pierdo puntos importantes porque estoy pensando en otras cosas” (Reversed) (item 33)

Cronbach alpha = 0.630

Elaboration

1.- When I study for this class, I pull together information from different sources, such as lectures, readings, and discussions/ “Cuando estudio, reúno información de diferentes fuentes como conferencias, lecturas y debates” (item 53)

2.- I try to relate ideas in this subject to those in other courses whenever possible/ “Intento relacionar las ideas de cada clase con las de otros cursos siempre que sea posible” (item 62)

3.- When reading for this class, I try to relate the material to what I already know/ “Cuando leo para mis clases trato de relacionar el material con lo que ya sé” (item 64)

4.- When I study for this course, I write brief summaries of the main ideas from the readings and the concepts from the lectures/ “Cuando estudio para mis clases escribo resúmenes de las ideas importantes” (item 67)

5.- I try to understand the material in this class by making connections between the readings and the concepts from the lectures / “Trato de comprender el material de mis clases hacienda conexiones entre las lecturas y los conceptos de las presentaciones” (item 69)

6.- I try to apply ideas from course readings in other class activities such as lecture and discussion/ “Intento aplicar las ideas de las lecturas de clase en otras actividades como conferencias y debates” (item 81)

Cronbach alpha = 0.821

Organization

1.- When I study the readings for this course, I outline the material to help me organize my thoughts/ “Cuando estudio para mis clases, bosquejo la información para ayudarme a organizar mis pensamientos” (item 32)

2.- When I study for this course, I go through the readings and my class notes and try to find the most important ideas/ “Cuando estudio para mis clases, reviso las lecturas y notas de clase y trato de encontrar las ideas más importantes” (item 42)

3.- I make simple charts, diagrams, or tables to help me organize course material/ “Realizo gráficos, diagramas o tablas simples para ayudarme a organizar la información” (item 49)

4.- When I study for this course, I go over my class notes and make an outline of important concepts/ “Cuando estudio para mis clases reviso mis notas y hago un resumen de los conceptos importantes” (item 63)

Cronbach alpha = 0.803

Intrinsic Goal orientation

1.- In a class like this, I prefer course material that really challenges me so I can learn new things/ “Prefiero que los trabajos de clase supongan un reto que me permita aprender cosas nuevas” (item 1)

2.- In a class like this, I prefer course material that arouses my curiosity, even if it is difficult to learn/ “Prefiero que la información de clases despierte mi curiosidad incluso si es difícil de aprender” (item 16)

3.- The most satisfying thing for me in this course is trying to understand the content as thoroughly as possible/ “Lo más satisfactorio para mí es entender los contenidos de clase de la mejor manera posible” (item 22)

4.- When I have the opportunity in this class, I choose course assignments that I can learn from even if they don't guarantee a good grade/ “Cuando tengo oportunidad de elegir, elijo hacer tareas en las que puedo aprender, aunque no me garanticen una buena nota” (item 24)

Cronbach alpha = 0.706

Supplementary File S3

Short version of the academic procrastination scale

Instructions: please indicate the degree of agreement or disagreement with the following statements**.** Answer options: 1 = absolutely disagree to 5 = absolutely agree.

(English/Spanish)

1.- I put off projects until the last minute/ "Dejo mis tareas hasta el ultimo momento"

2.- I know I should work on schoolwork, but I just don't do it/ "Sé que debería trabajar en mis tareas pero no lo hago"

3.- I get distracted by other, more fun, things when I am supposed to work on schoolwork/ "Me distraigo con otras cosas más divertidas cuando se supone que debería hacer tarea"

4.- When given an assignment, I usually put it away and forget about it until it is almost due/ "Cuando me dan una tarea, generalmente la guardo y me olvido de ella hasta que tengo el tiempo encima"

5.- I frequently find myself putting important deadlines off / "Frecuentemente me encuentro postergando trabajos/tareas importantes"

Cronbach alpha: 0.923
